# Chronic Δ9-tetrahydrocannabinol impact on plasticity, and differential activation requirement for CB1-dependent long-term depression in ventral tegmental area GABA neurons in adult versus young mice

**DOI:** 10.3389/fnins.2022.1067493

**Published:** 2023-01-09

**Authors:** Isaac Ostlund, Michael Von Gunten, Calvin Smith, Jeffrey G. Edwards

**Affiliations:** ^1^Department of Cell Biology and Physiology, Brigham Young University, Provo, UT, United States; ^2^Neuroscience Center, Brigham Young University, Provo, UT, United States

**Keywords:** LTD, VTA, development, CB1, THC, GABA, age

## Abstract

The ventral tegmental area (VTA) mediates incentive salience and reward prediction error through dopamine (DA) neurons that are regulated by local VTA GABA neurons. In young mice, VTA GABA cells exhibit a form of synaptic plasticity known as long-term depression (LTD) that is dependent on cannabinoid 1 (CB1) receptors preceded by metabotropic glutamate receptor 5 (mGluR5) signaling to induce endocannabinoid production. This LTD was eliminated following chronic (7–10 consecutive days) exposure to the marijuana derived cannabinoid Δ9 -tetrahydrocannabinol (THC). We now examine the mechanism behind THC-induced elimination of LTD in adolescents as well as plasticity induction ability in adult versus young male and female mice using whole-cell electrophysiology experiments of VTA GABA cells. Chronic THC injections in adolescents resulted in a loss of CB1 agonist-mediated depression, illustrating chronic THC likely desensitizes or removes synaptic CB1. We noted that seven days withdrawal from chronic THC restored LTD and CB1 agonist-induced depression, suggesting reversibility of THC-induced changes. Adult mice continue to express functional mGluR5 and CB1, but require a doubling of the synaptic stimulation compared to young mice to induce LTD, suggesting a quantitative difference in CB1-dependent plasticity between young and adult mice. One potential rationale for this difference is changes in AMPA and NMDA glutamate receptors. Indeed, AMPA/NMDA ratios were increased in in adults compared to young mice. Lastly, we performed quantitative reverse-transcription PCR and identified that CB1, DAGLα, and GluA1 levels increased following chronic THC exposure. Collectively, our data demonstrate the first age-dependent GABA neuron plasticity in the VTA, which could have implications for decreased THC dependence capacity in adults, as well as the mechanism behind chronic THC-induced synaptic alterations in young mice.

## Introduction

The ventral tegmental area (VTA) is an essential part of the mesocorticolimbic circuit of the brain, crucial for salience and reward-mediated learning and behavior. Dopamine (DA) neurons of the VTA project to structures regulating behavior including the nucleus accumbens (NAc) and prefrontal cortex (PFC) (Watabe-Uchida et al., 2012; Beier et al., 2015). DA projections from the VTA directly influence reward learning (Lisman and Grace, 2005; Ghanbarian and Motamedi, 2013), salience attachment to rewarding behaviors/stimuli (Lisman and Grace, 2005; Berridge, 2007), fear learning (Tang et al., 2020), reward or aversion (Gardner, 2005; Van Zessen et al., 2012; Bouarab et al., 2019), and reward prediction error (Schultz et al., 1997). Many of these learned responses in the VTA DA circuit depend on synaptic modifications as a result of experience (Koob and Volkow, 2010).

Synapses onto DA neurons undergo maladaptive drug-induced changes by inducing or preventing plasticity (Luscher and Malenka, 2012). These drug-induced synaptic plasticity changes are strongly correlated to drug dependence, as all addictive substances examined to date alter VTA glutamate plasticity onto DA cells, while non-addictive substances do not (Mameli and Luscher, 2011). These synaptic changes outlast the presence of drug in the system. Therefore, while drugs of abuse co-opt endogenous systems to increase DA release mediating reward (Robinson and Berridge, 1993), it is thought that drug dependence is caused by long-lasting synaptic changes resulting from adaptive cellular responses to the drug (Koob and Volkow, 2010). The term drug dependence here is used to indicate a pathological behavioral or physiological state created by chronic drug use that is synonymous with substance use disorder, often leading to withdrawal.

While DA neurons are the primary VTA cell type (∼65%), local GABA neurons make up a substantial population of the VTA (∼35% with ∼5% glutamatergic neurons) (Yamaguchi et al., 2007; Nair-Roberts et al., 2008). Some VTA neurons even contain enzymes for the production of both GABA and DA (Bouarab et al., 2019). These co-expressing neurons usually contain the GABA producing enzyme GAD65 (Gonzalez-Hernandez et al., 2001; Olson and Nestler, 2007), but not GAD67 (Merrill et al., 2015), which we use in our studies as a genetic marker for GABA neurons exclusively. GABA neurons innervate and regulate DA cell activity (Bocklisch et al., 2013) and exhibit plasticity that is modified by drugs of abuse (Bocklisch et al., 2013; Friend et al., 2017). These GABA neurons are both directly and indirectly involved in many forms of reward including consumption (Van Zessen et al., 2012), association and saliency (Brown et al., 2012), conditioned place preference, and aversion (Tan et al., 2012; Bocklisch et al., 2013). For example, DA levels can be indirectly increased by decreasing the activity of GABA neurons located either in the VTA (Edwards et al., 2017) or those projecting from other brain regions such as NAc (Ford et al., 2006; Bocklisch et al., 2013; Creed et al., 2014) and RMTg (Matsui and Williams, 2011). Synaptic GABA modulation of DA cells, alters DA release in areas such as the PFC and the NAc (Beier et al., 2015). VTA GABA neurons can also project directly to the NAc (Vanbockstaele and Pickel, 1995; Breton et al., 2019) where they can modify associative learning, reward reinforcement, and stress-induced blunted reward seeking (Brown et al., 2012; Al-Hasani et al., 2021; Lowes et al., 2021). Direct VTA GABA axonal projections to NAc cholinergic interneurons promote reward reinforcement (Al-Hasani et al., 2021), to midbrain are involved in opitate dependence (Ting et al., 2013), and to ventral pallidum encode reward value and motivation (Zhou et al., 2022).

VTA GABA cells also exhibit synaptic plasticity. We recently demonstrated a novel form of presynaptic glutamatergic LTD in juvenile/adolescent-aged mice dependent on cannabinoid 1 (CB1) receptors. This LTD is preceded by metabotropic glutamate receptor 5 (mGluR5) signaling to produce the endocannabinoid 2-arachidonyl-glycerol (2-AG) for retrograde presynaptic CB1 activation (Friend et al., 2017). Activation of CB1 results in presynaptic depression of excitatory transmission onto VTA GABA cells. The LTD was eliminated following chronic exposure to Δ9-tetrahydrocannabinol (THC), an exogenous cannabinoid extract from marijuana. As legislative bodies seek to implement laws regarding age of marijuana use and establish medical guidelines, examining THC’s mechanism of action and age-dependent changes within the VTA is essential. Compared to adults, adolescents are more susceptible to addiction caused by drugs of abuse (Chambers et al., 2003; Crews et al., 2007). For example, THC effects differ depending on the age of exposure both in humans and rodents (Mokrysz et al., 2016; Kasten et al., 2019). When pubescent rats are treated with a synthetic cannabinoid agonist, they show impairment in the novel object recognition task. If the rats are treated in adulthood, this impairment is not seen. However, the physiological mechanisms driving these age-dependent effects are less understood. Moreover, electrophysiological studies examining the effects of THC exposure on the VTA of adult models are almost non-existent. Because maladaptive drug-induced changes or dependence are now thought to involve alterations to synaptic plasticity, we used VTA GABA cell LTD as a model to examine this question. Therefore, we investigated differences in plasticity of young and adult mice and the mechanism and duration of THC-induced LTD elimination.

## Materials and methods

All experiments were performed in accordance with Institutional Animal Care and Use Committee protocols and followed National Institutes of Health Guide for the care and use of laboratory animals. All experiments were approved by the Brigham Young University Institutional Animal Care and Use Committee, Animal Welfare Assurance Number: A3783-01.

### Animals

Male and female juvenile/adolescent (P15–P40) or adult (P70-P150) aged CD1 GAD67-GFP knock-in mice produced by the Tamamaki laboratory were used so that GABA cells in the VTA could be positively identified using fluorescence (Tamamaki et al., 2003). Juvenile age in mice begins at ∼P14–P15 and adolescence begins at ∼P28–P35 (Spear, 2000; Crews et al., 2007; Iniguez et al., 2016; Liu et al., 2017; Yohn and Blendy, 2017). No statistical differences in LTD were noted between juvenile and adolescent mice and therefore they were grouped as “young” animals in analysis. Early adulthood begins at P60-P90 (Stanwood et al., 1997; Spear, 2000; Yu et al., 2006). No difference was observed between males/females within the experimental groups and they were also grouped together in analysis. Glutamate decarboxylase (GAD65/GAD67) is required to convert glutamate to GABA and is a key identifier of GABA neurons. Cells expressing GAD65 also express tyrosine hydroxylase, a DA releasing neuron marker, suggesting a subset of neurons co-release GABA and DA (Bouarab et al., 2019). However, cells expressing GAD67 comprise the largest subset of GABA neurons within the VTA and do not co-express DA neuron proteins such as TH (Merrill et al., 2015). For supplemental data male and female Sprague-Dawley rats (P23-45) were used to examine LTD occurrence in another animal model to confirm LTD is a conserved form of plasticity. Rats were acquired from Charles River laboratory and handled identically to mice in these electrophysiology experiments.

### Whole tissue and single cell qRT-PCR

For qPCR experiments we used VTA samples from mice that were separated into five treatment groups of either young (P15-32) or adult (P70-150): naive young, young mice chronically exposed to THC, young mice chronically exposed to THC with a seven-day withdrawal, chronic ethanol vehicle injected mice, or naive adult. The mRNA from each of these mice were collected by tissue punches of VTA taken from 400 μm thick brain slices. Samples of mouse VTA were temporarily stored at +4°C and extracted using TRIzol solution (Invitrogen, Carlsbad, CA) per a modified chloroform phenol extraction method (Toni et al., 2018). Efficacy of each step was measured for both quantity and purity using a spectrophotometer. After mRNA extraction, reverse transcription was performed on each sample according to iScript Supermix (BioRad) protocol. The iScript Supermix protocol was performed in a C1000 Thermocycler (BioRad) and was: 25.0°C for 8 min, 42.0°C for 60 min, and 70°C for 15 min. After this process, the mRNA was successfully converted to create a cDNA library of each mouse VTA. In order to ensure specificity in qRT-PCR, we carefully design primers for each molecular target using the NCBI Primer-Blast database and subjected them to a 5 concentration serial dilution quantitative PCR test to determine efficiency and recorded melting temperature. All newly designed and previously published primers span exon-exon boundaries as described previously (Merrill et al., 2012), except CB1, which lacks introns. Primers had an efficiency of 90–100%. All the primers were purchased from Invitrogen. Table 1 displays each target’s gene name, protein name, forward and reverse primer sequence and its respective amplicon size. After each primer was tested, the VTA cDNA libraries from each group underwent quantitative PCR, where each sample was placed into reaction wells in triplicate of a Bio Rad CFX96 quantitative PCR machine (BioRad) using Sso Fast EvaGreen Supermix (BioRad) according to the following protocol: 95°C hot start for 3 min, followed by 60 cycles of 95°C for 15 s, 57°C for 20 s, and 72°C for 25 s. Melting temperature and gel electrophoresis of amplicons were analyzed to ensure specificity of the DNA amplicon. Amplification was measured by increased relative fluorescence during each cycle and a cycle threshold (Ct) value was assigned to each target using BioRad CFX Manager Software. Cycle threshold data analysis was carried out as described previously (Merrill et al., 2015). Gels of amplicon for primers sets not shown here were published and verified previously (Merrill et al., 2015; Miller et al., 2018).

**TABLE 1:** qRT-PCR targets, primers, and amplicon sizes.

Name	Protein name	Forward	Reverse	Amplicon
18s	Ribosomal Protein S18	GTGCATGGCCGTTCTTAGTTG	GCCACTTGTCCCTCTAAGAAGTTG	133bp
BDNF	Brain Derived Neurotrophic Factor	GACATCACTGGCTGACACTTTTG	CAAGTCCGCGTCCTTATGG	100 bp
CB1	Cannabinoid Receptor 1	TGAGGTCCTGGCAATGAGCA	GTGACTGAGAAAGAGGTGCCA	145bp
COMT	Catechol-O-Methyltransferase	CTTGACGAGGGGATGAGAGAGT	CAATGAGACAGCAGCCAACAG	90bp
CREB	CREB Binding Protein	TGGAAGAACTGCACACGACA	ATTCTGTTGCCCTGCACCAA	144bp
DAGLα	Diacylglycerol Lipase Alpha	AGAAGAAGTTGGAGCAGGAGATG	AAGGAGTGGCCTACCACAATC	100bp
EGR1	Early Growth Response 1	TATGCTTGCCCTGTCGAGTC	GATGCGGATATGGCGGGTAA	72 bp
FAAH	Fatty Acid Amide Hydrolase	AGGATTTGTTCCGCTTGGACT	AGTGGGCATGGTGTAGTTGT	119bp
GAPDH	Glyceraldehyde-3-Phosphate Dehydrogenase	CCCCATGTTTGTGATGGGTGT	AGCCCTTCCACAATGCCAA	138bp
GluA1	Glutamate Ionotropic Receptor AMPA Type Subunit 1	CAAGGAACTGCAGGAAGAAA	CTAGAAAACCGGTGCAGAAA	157bp
GluA2	Glutamate Ionotropic Receptor AMPA Type Subunit 2	GAGGAAGAAAGGGAAACGAG	TCAGTCCCCATAAAACAGGA	109bp
GluN1	Glutamate Ionotropic Receptor NMDA Type Subunit 1	CCACGAGCTCCTAGAAAAGG	TCTGCATACTTGGAAGACATCAG	120bp
GluN2a	Glutamate Ionotropic Receptor NMDA Type Subunit 2A	CGCTCTGCTCCAGTTTGTTG	GCTGCTCATCACCTCATTCTTC	100bp
GluN2b	Glutamate Ionotropic Receptor NMDA Type Subunit 2B	TGGTATCACGCAGCAATGG	CAGAGACAATGAGCAGCATCAC	102bp
HDAC3	Histone Deacetylase 3	CCCCCTTTCCTCAAACTCTC	TTGCATGGAAGCAAGAACTG	238bp
MAGL	Monoglyceride Lipase	CCAGGCGAACTCCACAGAAT	TGGGACACAAAGATGAGGGC	127bp
mGluR1	Glutamate Metabotropic Receptor 1	ATATCGTCAAGCGGTACAACTG	GGCAGCCAACTCTTTGAAAG	98bp
mGluR5	Glutamate Metabotropic Receptor 5	CTGCACACCTTGTAAGGAGAATG	CAAATCACAACCTGTCAAGTCG	103bp
NR4A2	Nuclear Receptor Subfamily 4 Group A Member 2	TCTCCTGACTGGCTCTATGG	AGCAAAGCCAGGAATCTTCT	61bp
PER1	Period Circadian Regulator 1	GAAACGGCAAGCGGATGG	GATGGGACTCCTCCAGGACA	250bp

Relative quantities of gene expression were determined using Microsoft Excel and the Livak and Schmittgen ΔΔCt/Cq method (Livak and Schmittgen, 2001). Cycle threshold data was then averaged and analyzed according to ΔΔCt analysis. Both 18S ribosomal and GAPDH were averaged to function as a transcription baseline (Riedel et al., 2014). The resulting ΔΔCt values for each target were compared using SPSS statistics software by one-way ANOVA comparing between each treatment group (Livak and Schmittgen, 2001; Yuan et al., 2006). Statistical analysis was chosen to be done at the level of ΔΔCt and 2^ΔΔ*Ct*^ to avoid potential bias toward a significant relationship (Yuan et al., 2006). A *post hoc* Tukey test was analyzed to compare significance between treatment groups. Data was compiled and presented as a ratio of 2^ΔΔ*Ct*^ normalized to naive young mice.

Single cell qRT-PCR was performed as above with the following exceptions. Single cells, and their ACSF controls from the same slice and bath solution, were pulled and immediately underwent the iScript reverse transcriptase protocol. The targets were TH, GAD67, and 18S. TH reactions used the appropriate FAM-TAMRA probe (Applied BioSystems, Inc.) while 18S and GAD67 reactions used Sso Fast EvaGreen Supermix with the correlating melt curve analysis to ensure target specificity. The TH probe sequence was: TCTCGTATCCAGCGCCCATTCTC. GAD67 rat primer sequences were: forward, ATCATGGCT GCTCGTTACAAGTAC; reverse, AATAGTGACTGTGTTCTG AGGTGAAG. TH and 18S primer sequences and gel were published previously (Merrill et al., 2012). A 15 cycle multiplex reaction of all primer targets took place before quantitative PCR measurements using our multiplex protocol (Merrill et al., 2015) on a C1000 Thermocycler (BioRad) that was: 95.0°C hot start for 3 min, followed by 15 cycles of 95.0°C for 15 s, 57°C for 20 s, and 72°C for 25 s. The quantitative PCR measurements for single cells were conducted using a BioRad CFX96 with the same protocol as our whole tissue cDNA libraries. Ct value data from the quantitative PCR reaction from each cell was compared to Ct data from control artificial cerebrospinal fluid samples extracted from each slice. Proper amplification of each cellular target was also examined using 4% agarose gel electrophoresis to verify amplicon size.

Targets for PCR were created in three categories to examine changes in glutamate receptor subtypes known to be involved in plasticity, endocannabinoid elements, or known modifiers of epigenetics or development. Glutamate receptors examined included AMPA (GluA1, GluA2), NMDA (GluN1, GluN2a, GluN2b) or metabotropic (mGluR1, mGluR5) subtypes. Endocannabinoid components included the cannabinoid receptor (CB1), which is directly targeted by THC, enzymes involved in 2-AG synthesis (DALα) and degradation (MAGL), or anandamide degradation (FAAH). Anandamide is the second major endocannabinoid along with 2-AG, and has multiple synthesis enzymes. Elements involved in genetic regulation (epigenetics), development, etc. were BDNF, a growth factor, COMT, known to modulate plasticity, CREBBP modulator of transcription, ERG1 a transcription factor, HDAC, a histone deacetylase, NR4A2 a nuclear receptor and PER1 an epigenetic regulator, among other functions. Chronic drug exposure is well known to alter epigenetic factors such as these that could lead to drug-induced behavioral changes in rodents and humans.

### Slice preparation

Mice were anesthetized with isoflurane and decapitated with a rodent guillotine. Brains were rapidly removed and sectioned horizontally on a vibratome at 250 μm. Young brains were sliced using an ice-cold, sucrose-based cutting solution composed of 220 mM sucrose, 0.2 mM CaCl_2_, 3 mM KCl, 1.25 mM NaH_2_PO_4_, 25 mM NaHCO_3_, 12 mM MgSO_4_, 10 mM glucose, and 400 μM ascorbic acid. After sectioning, the slices were placed in oxygenated ACSF composed of 119 mM NaCl, 26 mM NaHCO_3_, 2.5 mM KCl, 1 mM NaH_2_PO_4_, 2.5 mM CaCl_2_, 1.3 mM MgSO_4_, and 11 mM glucose in an incubator at 35°C for an hour and then transferred to room temperature until recording identical to our prior publication in adolescents (Friend et al., 2017). To better preserve adult VTA slices, adult brains were sliced using an ice-cold N-methyl-D-glucamine (NMDG)-based cutting solution composed of 92 mM NMDG, 2.5 mM KCl, 1.25 mM NaH_2_PO_4_, 30 mM NaHCO_3_, 20 mM HEPES, 25 mM glucose, 2 mM thiourea, 5 mM Na-ascorbate, 3 mM Na-pyruvate, 0.5 mM CaCl_2_⋅2H_2_O, and 10 mM MgSO_4_⋅7H_2_O, titrated to 7.5 pH with HCL. Cardiac perfusions were performed in adult mice using 10 mL ice cold, oxygenated NMDG cutting solution that was injected into the left ventricle of the heart directly before decapitation and brain removal. After sectioning, adult slices were kept in a warm (34°C) bath of the cutting solution and were spiked with increasing amounts of NaCl over 20 min before being placed in an oxygenated HEPES holding solution composed of 92 mM NaCl, 2.5 mM KCl, 1.25 mM NaH_2_PO_4_, 30 mM NaHCO_3_, 20 mM HEPES, 25 mM glucose, 2 mM thiourea, 5 mM Na-ascorbate, 3 mM Na-pyruvate, 2 mM CaCl_2_⋅2H_2_O, and 2 mM MgSO_4_⋅7H_2_O titrated pH to 7.5 with 10 N NaOH, as described by Ting et al (Ting et al., 2018).

### Recording protocol experimental design

Recordings began at least 1 h after cutting. Slices were placed in the recording chamber and bathed with oxygenated (95% O2, and 5% CO2) high divalent ACSF (4 mM CaCl_2_ and 6 mM MgSO_4_), along with 100 μM picrotoxin to block GABA_*A*_ currents. High divalent cation solution is used to prevent action potentials in GABA cells that normally sit closer to threshold than other neurons, which avoids voltage-channel currents overlapping synaptic currents. We and other labs has used this technique previously and note no changes to plasticity induction (Gibson et al., 2008; Friend et al., 2017). Experiments were performed at an average temperature of 29.4°C. The VTA was visualized using an Olympus BX51W1 microscope with a 40× water-immersion objective. GAD67-GFP-positive cells were selected for recording from approximately the following coordinates from adult mouse bregma; anteroposterior -2.9 to -3.1, mediolateral 0.1 to 0.4, dorsoventral -3.9 to -4.1. Patch pipette resistance was 2.5–6.5 M. Distances between recording electrode and stimulating electrode were between ∼150 and 350 μm. Distances limited to ∼150–250 μm of recorded cells were used for data analysis of input/output curves for more accurate comparison between adults and adolescents. Bipolar concentric stimulating electrodes (Microprobes) were employed to evoke excitatory postsynaptic currents (EPSCs). Cells were held at -65 mV and patched with a glass pipette filled with internal solution composed of 117 mM cesium gluconate, 2.8 mM NaCl, 20 mM HEPES, 5 mM MgCl_2_, 2 ATP-Na, 0.3 GTP-Na, 0.6 μM EGTA-K and 1 mM QX-314 (Tocris Bioscience), pH 7.28 (275–285 mOsm). Current traces were recorded using Multiclamp 700B amplifier (Molecular Devices). Signals were filtered at 4 kHz and digitized with an Axon 1550A digitizer (Molecular Devices) connected to a Dell personal computer with pClamp 10.7 Clampex software (Molecular Devices). Sampling frequency was 0.1 Hz. The conditioning stimulus to induce plasticity was a high frequency stimulus (HFS) of two, one-second duration 100 Hz bursts, separated by 20 s. The peak amplitude of the induced EPSC was calculated using Clampfit 10.7 software (Molecular Devices) and graphed using Origin 7.5. The cell input resistance and series resistance were monitored continuously throughout each experiment by 5 mV, 100 ms hyperpolarization pulses; cells were discarded if these values changed by more than 15% during the experiment. Within individual experiments, current amplitudes were averaged by minute (6 sweeps/min).

When generating AMPA/NMDA ratios and IV plots, spermine (0.1 mM) was included in the internal solution to avoid polyamine dialyzation and maintain its block of GluA2-lacking AMPA receptors at depolarized potentials, thereby determining the subunit composition of synaptic AMPA receptors. To obtain the AMPA/NMDA ratio, cells were first held at -70 mV during whole cell recording. Prior to the recording, at least 10 min was given for spermine to dialyze into the cell; the holding potential was then raised to + 40 mV to allow for recording of NMDA currents, which are blocked by magnesium at negative holding potentials, and then baseline EPSCs were recorded for several minutes Next at +40 mV. APV (50 μM) was then bath applied to block NMDA receptors and thus isolate AMPA-only currents. NMDA currents were determined by digital subtraction of AMPA-only currents from baseline EPSCs made up of both AMPA and NMDA currents. AMPA and NMDA ratios were attained by dividing the two and noting changes in ratio level, illustrating changes in synaptic glutamate receptor. Immediately after the AMPA currents were recorded, the holding potential was moved back gradually from + 40 to -70 mV in 20 mV steps in order to create IV plots. The IV plot can be used at positive potentials to exam inward rectification, which is common when changes to AMPA receptor subunits occur, in particular to the GluA2 subunit. The -70/ + 40 ratio was calculated based on the average currents at these holding potentials. Traces are recording overlays compiled from progressively increasing voltage inputs. Similar to IV plot, this ratio examines changes in AMPA receptor subunits. Input-output curves were created by *post hoc* analysis of raw data using two-minute averages of EPSCs comparing the amount of current evoked in an AMPA or NMDA only EPSC at + 40 mV compared to current injected via the stimulation electrode. Note the distance between the stimulating electrode and cell were kept within a consistent range for accurate young versus and adult AMPA and NMDA input/output comparison.

Analysis for paired-pulse ratios (PPRs) and coefficient of variance (1/CV^2^) are calculated from raw data as described previously (Gibson et al., 2008). In all experiments a second stimulus was given after a 50 ms delay to create a second EPSC. The PPR ratio was calculated by dividing a five minute average of the second peak response by a five minute average of first peak response, taken the last five minutes of baseline and 10–15 min post-conditioning. PPR is used as a suggestive measure of plasticity location (presynaptic versus postsynaptic). The 1/CV^2^ is proportional to release probability and is also suggestive of plasticity location. The 1/CV^2^ is calculated using raw data and determined using successive epochs of 30 trials taken of the last five minutes of baseline and 10–15 min post-conditioning as 1/CV^2^ = [μ/σ]^2, where σ = the standard deviation of epochs and μ = the mean.

### Solutions and pharmacological agents

Picrotoxin (100 μM) was purchased from Abcam or Tocris Bioscience. APV (50 μM), AM251 (2 μM), DHPG (100 μM), and WIN55,212-2 (10 μM), were purchased from Tocris Bioscience. Spermine was purchased from Sigma. THC was provided by the National Institute on Drug Abuse Drug Supply Program. THC was shipped to us dissolved in ethanol and was administered via intraperitoneal injection at 10 mg/kg, with a final injection of volume of 50 μl. Recordings began 24 h following the final injection. For chronic THC injections mice were injected daily and processed as cohorts for 7–10 days, where this day spread accounted for the weekend and/or the variability in the exact day each mouse in the cohort was used. Vehicle injected controls included the same concentration of EtOH, which THC was diluted in. All stock solutions were frozen at -20°C; the final concentration was attained by dilution in ACSF for THC bath application or saline for THC injections.

### Statistics

A *p* value of <0.05 was considered significant and listed as such. For experiments where the *p* value was much smaller, we used *p* < 0.001 in order to share as much information as possible with the reader, and if not significant we used *p* > 0.1. Trends (0.1 > *p* > 0.05) in significance were not used to draw conclusions, but were included in the paper for interpretation purposes by adding more context to the data analysis. For example, a trend suggest increasing the experimental variable could potentially alter the results, which can in some cases be useful data to present. ANOVAs were used to compare for differences within a single experiment to determine if plasticity was significantly different compared to baseline, as well as for quantitative PCR experiments. ANCOVA was used to examine changes in IV and input/output curve data. An unpaired two-way student’s T test was used to compare between different groups of experiments either post-conditioning or post-drug application. Statistics for electrophysiology compared the last five minutes of baseline to another five minute range at 15–20 min post-conditioning or post-drug application using an ANOVA. A Wilcoxin ranked-sum test was used for paired pulse ratio and coefficient of variance analysis. This type of test for PPR and 1/CV^2^ is common as the range of data is so great, and not normalized. Differences were considered significant at *p* < 0.05, except non-ratio patch-clamp experiments which were subject to a Bonferroni correction and considered significant at *p* < 0.002. To control for multiple comparisons, a simple Bonferroni correction was implemented in electrophysiological analysis. A total of 24 comparisons for whole-cell patch plasticity experiments were performed, and therefore a critical level for significance of 0.05/25 (0.002) was used. No differences were noted between males and females during data analysis.

## Results

Confident identification of VTA GABA neurons for electrophysiological experiments was vital to this study as they can be confused with DA neurons. Therefore, GAD67-GFP mice were used for GABA cell identification, as the majority of VTA GABA neurons express glutamic acid decarboxylase 67 (GAD67) and DA neurons do not (Beier et al., 2015; Merrill et al., 2015). Therefore, when we refer to GABA neurons, we are referring to GAD67 + neurons. Whole cell electrophysiology was used to record evoked EPSCs for these experiments.

**FIGURE 1:**
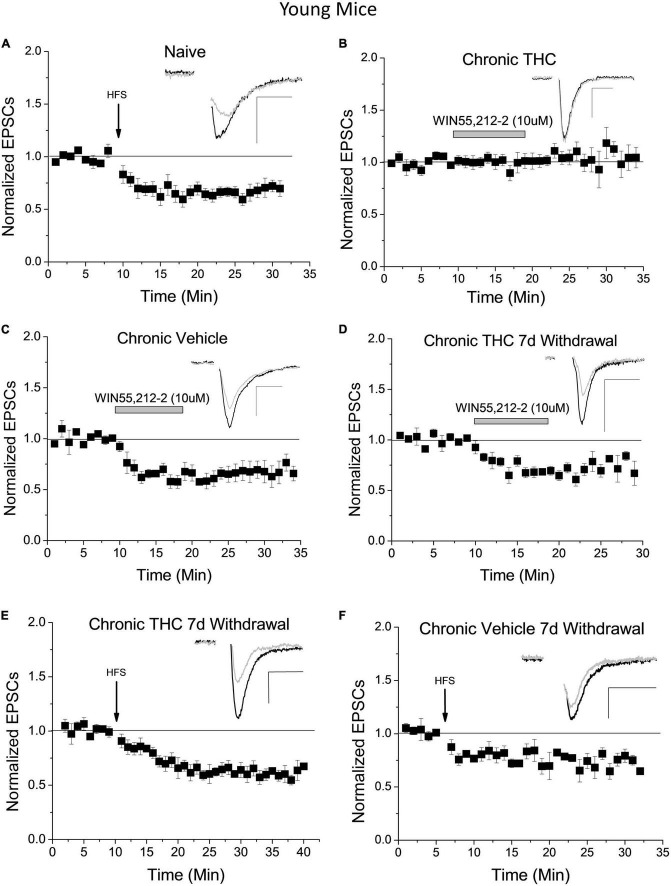
Chronic THC impact on glutamate inputs and plasticity of VTA GABA neurons. **(A)** Within naïve young mice (P15-40) a high frequency stimulus (HFS) induces LTD (33 ± 7.1% reduction) 15–20 min post stimulus compared to baseline (*n* = 7, *p* < 0.001), confirming previously reported LTD in mice (Friend et al., 2017). **(B)** Young mice with chronic (7–10 consecutive days) THC intraperitoneal (IP) injections do not exhibit WIN55,212-2-induce depression compared to baseline (*n* = 8, *p* > 0.1). **(C)** In contrast, young mice chronically injected with vehicle (0.1% EtOH saline) exhibit significant WIN55,212-2-induced depression compared to baseline (38.3% ± 6.3% depression, *p* < 0.001). **(D)** Seven days withdrawal following chronic exposure to THC in young mice, WIN55,212-2 again induced depression of excitatory currents (by 26.3 ± 7.3%; *n* = 6, *p* < 0.001), which is significantly different than the chronic THC group in panel **(B)** at the same time points (*p* < 0.001; 15–20 min post-drug application). **(E)** Chronic THC elimination of HFS-induced LTD is reversed in young mice by seven days withdrawal as they undergo LTD (38.1 ± 5.7%; *n* = 6, *p* < 0.001). This is not significantly different (*p* > 0.1) than naïve LTD in panel **(A)**. **(F)** HFS continues to induce significant LTD within age-match control mice chronically injected with vehicle after 7 days withdrawal (29.3 ± 7.3%; *n* = 4, *p* < 0.001). Here and throughout horizontal gray bars indicate duration of drug application (10 min) and whole-cell plots are normalized excitatory post-synaptic currents (EPSC) amplitude means with bars indicating standard error of the mean (SEM). All recordings were excitatory glutamate currents recorded from GAD67/GFP + neurons. *N* values are number of individual cells, with one cell per animal recorded. Arrows indicate time of HFS. Example traces representing 12–14 averaged traces taken before (black) and 15–20 min after (gray) conditioning or drug application. Note that all stimulation artifacts were removed from example traces for clarity. Scale bars here and throughout represent 50 pA, 5 ms. Supplementary Table 1 is a list of statistics and significance values of all the experiments in these figures.

We previously reported (Friend et al., 2017) and confirm here that naïve young mice undergo LTD of excitatory inputs to GABA neurons within the VTA following high-frequency stimulation (HFS; Figure 1A). To determine whether HFS-induced LTD is a form of synaptic plasticity conserved between species, and thus potentially an important form of plasticity for normal VTA GABA cell function, we performed experiments in the rat VTA. LTD of glutamatergic synapses onto VTA GABA cells occurs in the young rat, similar to the mouse (Supplementary Figure 1A). Rat GABA neurons were identified using single-cell PCR and by the absence of an I_*h*_ current (Supplementary Figure 1B). Quantitative RT-PCR confirmed GAD67 expression and the absence of tyrosine hydroxylase in experimental rat cells. While I_*h*_ is not an infallible cell type indicator, this taken together with PCR results is a reliable determinant of rat GABA cells.

In addition, we previously reported that the CB1 agonist WIN55,212-2 depressed glutamatergic input to naïve VTA GABA neurons and that HFS-induced CB1 receptor-dependent LTD of these neurons is lost following chronic THC injection in young mice (Friend et al., 2017). However, the mechanism of LTD elimination by THC is unknown. It is possible that chronic THC exposure reduced synaptic CB1 receptor expression (Puighermanal et al., 2013) or altered production capacity or degradation of the eCB 2-AG. To differentiate between these hypotheses we applied WIN55,212-2, a CB1 receptor agonist, to brain slices of young mice chronically (7–10 consecutive days) intraperitoneal (IP) injected with THC (10 mg/kg). Within the chronic THC injected group, WIN55,212-2-induced depression was eliminated (Figure 1B). This elimination suggests that prolonged exposure to THC either reduces CB1 receptor expression, desensitizes CB1 receptors, or alters a downstream signaling pathway. Note this does not exclude the possibility that enhanced degradation or reduced production of 2-AG could also be occurring, which is addressed below. Control vehicle-injected animals continue to exhibit WIN55,212-2-induced depression (Figure 1C). Next, we examined reversal of chronic THC impact at these synapses by performing WIN55,212-2 bath experiments in young mice chronically THC-injected 7 days post-drug cessation, hereafter designated withdrawal. After withdrawal, chronic THC elimination of WIN55,212-2 induced synaptic depression is reversed (Figure 1D). This suggests CB1 function and/or 2-AG levels are restored to naïve conditions. HFS-induced LTD is also restored following 7 days of THC withdrawal (Figure 1E). Chronic vehicle experiments with age-matched controls also continue to exhibit LTD after a 7-day withdrawal (Figure 1F).

**FIGURE 2:**
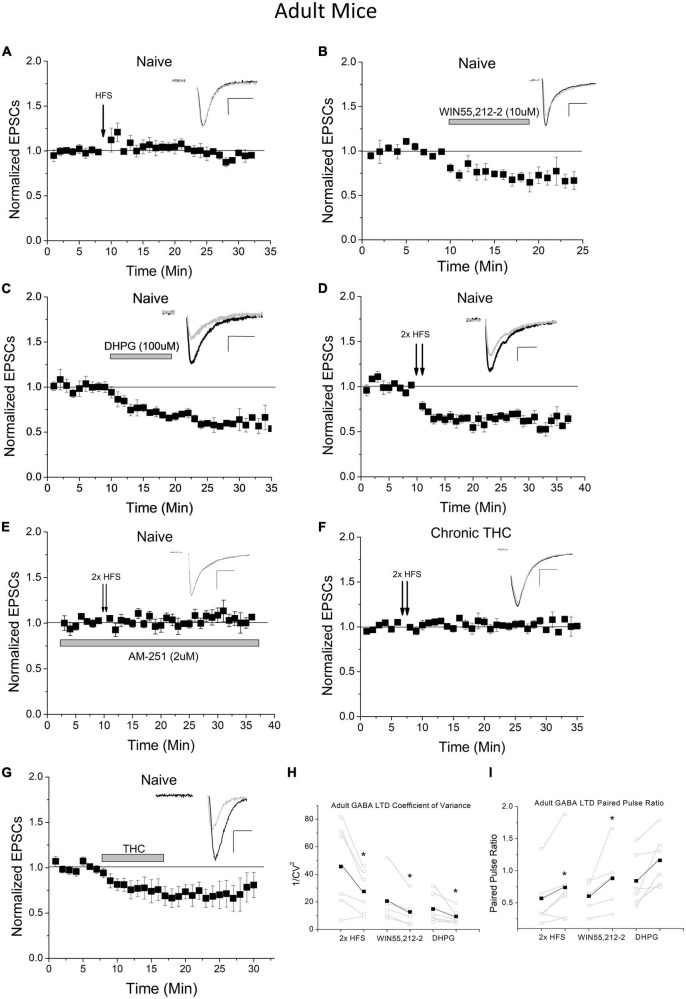
Adult mice exhibit increased LTD induction thresholds of excitatory inputs to VTA GABA neurons. **(A)** HFS does not induce LTD in adult mice compared to baseline (1.4 ± 5.7%, *n* = 8, *p* > 0.1, P70-150) and is significantly different than HFS young mice at the same time points compared to Figure 1A (*p* < 0.001). **(B)** WIN55,212-2 significantly depresses excitatory currents in adult mice compared to baseline (by 31.0 ± 10.1%; *n* = 5, *p* < 0.001) and is significantly different than the naïve adult HFS group at the same time points (*p* < 0.001). **(C)** DHPG significantly depresses excitatory postsynaptic currents (EPSCs) in adult mice compared to baseline (by 41.7 ± 3.1%; *n* = 6, *p* < 0.001) and is significantly different than the naïve adult HFS group at the same time points (*p* < 0.001). Note that WIN55,212-2 and DHPG-induced depression in adults are not significantly (*p* > 0.1) different from depression induced by these agonists in adolescents, comparing to data in our prior publication (Friend et al., 2017). **(D)** Doubling the HFS protocol induces LTD in adult mice compared to baseline (32.3 ± 4.4%; *n* = 6, *p* < 0.001) and is significantly different than the naïve adult HFS group at the same time points (*p* < 0.001). **(E)** The CB1 antagonist AM-251 (2 μM) blocked 2× HFS LTD (*n* = 6, 4.3 ± 2.2% above baseline, *p* > 0.1), which was not significantly different (*p* > 0.1) than control HFS in panel **(A)**. **(F)** Chronic (7–10 days) THC exposure results in a loss of 2× HFS-induced LTD in adult mice (*n* = 6, *p* > 0.1) and is significantly different than naïve adults with double HFS (*p* < 0.001). This is comparable to chronic THC elimination of HFS-induced LTD seen in young mice (see Friend et al., 2017). **(G)** Application of THC (1 μM) to the bath ACSF induces significant depression (*p* < 0.001) compared to baseline (35% depression), which was not significantly (*p* > 0.1) different from THC-induced depression in adolescents (20% depression (Friend et al., 2017). **(H)** The coefficient of variance (1/CV^2^) in adult experimental groups showed a significant decrease following 2× HFS LTD (*n* = 6, *p* < 0.05), WIN55,212-2-induced depression (*n* = 5, *p* < 0.05), and DHPG-induced depression (*n* = 6, *p* < 0.05). **(I)** The paired pulse ratio (PPR) for adult experimental groups increased when comparing baseline to post 2× HFS LTD (*n* = 6, *p* < 0.05) and WIN55,212-2-induced depression (*n* = 5, *p* < 0.05). No significant change was noted following DHPG depression, but trend only (*n* = 6, 0.1 > *p* > 0.05).

As the negative long-term impact of THC in humans is associated more with adolescents than adults, and because the endocannabinoid system and CB1 receptor expression changes into adulthood compared to adolescents, we examined whether adult mice exhibit the same plasticity as young mice. We performed HFS experiments in VTA GAD67+ neurons of adult (P70-P150) animals and determined that adults do not exhibit HFS-induced LTD at excitatory inputs onto GABA neurons (Figure 2A). To investigate the absence of HFS-induced LTD in adult mice, we assessed the functionality of CB1 and mGluR5 at this synapse. WIN55,212-2 was applied to naïve adult brain slices while recording VTA GABA cells to examine CB1 functioning. WIN55,212-2 continues to depress glutamate synapses in adults, which was not significantly different (*p* > 0.1) from young mice (Figure 2B; Friend et al., 2017), suggesting synaptic CB1 receptor is present and functional. As CB1-dependent LTD requires mGluR5 activation to induce 2-AG formation in adolescent mice, type I mGluR (mGluR1/5) agonist DHPG was applied to naïve adult slices. DHPG continues to induce depression of neurotransmission at this synapse (Figure 2C), again that was not significantly (*p* > 0.1) different from young mice (Friend et al., 2017). Since all LTD induction mechanisms are present in adults, we examined next whether LTD in adults could be quantitatively-different and thus require a larger stimulus to induce activation. To clarify, a qualitative difference in plasticity would be a unique signaling mechanism, while a quantitative difference would use the same mechanism but require a different degree of activation. To do this we doubled the HFS protocol. We noted that this greater stimulation induced LTD in adults to a similar degree as a single HFS in young animals (Figure 2D). This illustrates that the discrepancy in LTD induction in adults is quantitative in nature. To confirm whether LTD was qualitatively similar to adolescents we investigated whether this LTD was CB1-dependent. We determined that 2 x HFS induced LTD is blocked by application of the CB1-receptor antagonist AM-251 (Figure 2E), illustrating this plasticity is CB1 receptor-dependent, similar to HFS-induced LTD in young mice (Friend et al., 2017). We next sought to determine whether chronic THC can eliminate this plasticity as it does in adolescents. We chronically injected adult mice with THC for 7–10 days and performed our double HFS protocol. We determined that chronic THC injection also eliminated double HFS-induced LTD of adult VTA GABA neurons (Figure 2F). Bath application of THC (1 μM) also induced synaptic depression, demonstrating an acute impact on THC on synaptic transmission in adults (Figure 2G). Both coefficient of variation analysis (Figure 2H) and pair pulse ratios (Figure 2I) of evoked EPSCs from 2× HFS LTD, as well as DHPG and WIN55,212-2 agonist-induced depression, suggest adult LTD/depression is presynaptic, as previously noted in young mice (Friend et al., 2017).

As a note, we performed control experiments to ensure that the cutting ACSF had no effect on plasticity, as adolescent mice brains were cut in high sucrose ACSF and adults in NMDG solution. No effect of cutting solution was noted when these were reversed as young mice still undergo LTD following HFS when cut in NMDG solution (Supplementary Figure 2A), and adults continue to not exhibit LTD after slicing in sucrose (Supplementary Figure 2B).

**FIGURE 3:**
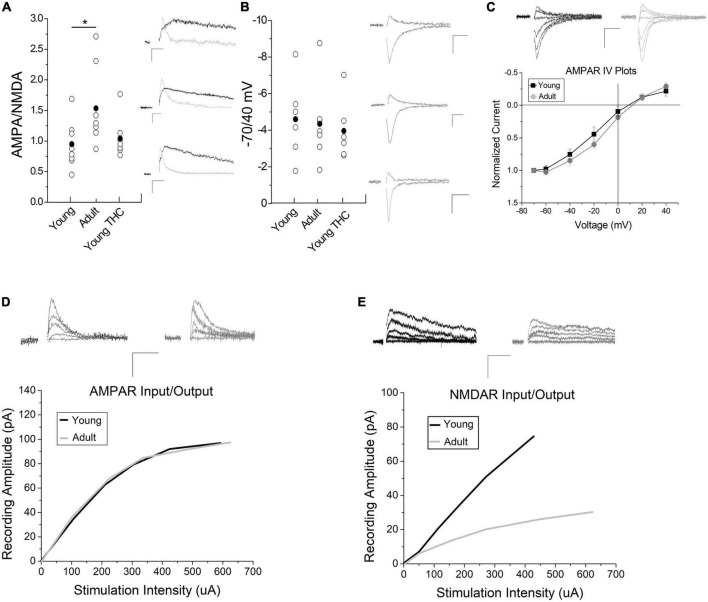
Impact of development and chronic THC on AMPA/NMDA ratios. **(A)** AMPA/NMDA ratios are significantly higher in adult mice than young (*n* = 8 young, *n* = 7 adults, *p* < 0.05). Chronic THC injection in young mice has no significant effect on AMPA/NMDA ratios when compared to naive young mice (*n* = 7 young chronic THC, *p* > 0.1). Insets represent AMPA currents and NMDA currents at +40 mV, the latter being a subtraction of AMPA from total current (see methods for details). **(B)** The -70/40 ratio remains unchanged from naive young mice to adults (*n* = 6 young and *n* = 7 adult, *p* > 0.1), and from naïve young mice to chronic THC injected young mice (*n* = 6 chronic THC young, *p* > 0.1). Insets represent AMPA currents taken at -70 and +40 mV. **(C)** No significant difference is noted in IV plots between young and adult mice (*n* = 7 for both groups, *p* > 0.1; ANCOVA). Rectification between the two groups at positive potentials is unchanged (*p* > 0.1). Insets represent AMPA currents taken at +40, +20, 0, -20, -40, -60, and -70 mV. **(D)** Input/output curves of evoked AMPA currents as correlated with stimulation intensity were unchanged between age groups (*n* = 15 adults, *n* = 13 young, *p* > 0.1, ANCOVA). Curves were created by *post hoc* analysis of raw data using two-minute averages of EPSCs comparing the amount of current evoked in an AMPA or NMDA only EPSCs at +40 mV compared to current injected into the stimulation electrode, which were then best fit to a curve. The data points used to create the curves were taken at various points throughout the curve range. The curves were compared statistically between young and adult mice for AMPA **(D)** and NMDA **(E)** currents using an ANCOVA. Raw data was that attained in Figure 3A at +40 mV in order to measure NMDA currents. **(E)** Input/output curves of evoked NMDA currents were significantly higher in young mice compared to adults (*n* = 9 adults, *n* = 8 young mice, *p* < 0.05, ANCOVA). In both panels **(D,E)** inset AMPA and NMDA current traces were measured at +40 mV at various stimulus intensities. Scale bars represent 20 pA, 5 ms. *p < 0.05.

Collectively, we determined that electrically evoked LTD is qualitatively similar in young and adult animals (i.e., the same mechanism), and we know that this LTD requires NMDA receptor activation (Friend et al., 2021). Thus we hypothesized that changes in synaptic NMDA receptor quantity could result in plasticity differences between adult and young mice. Therefore, we performed AMPA/NMDA ratio experiments and determined that the AMPA/NMDA ratio is significantly (*p* < 0.05) increased in adults compared to young mice (Figure 3A). An increased AMPA/NMDA ratio could imply either increased levels of AMPA receptors, changes in AMPA receptor subtype, or decreased levels of NMDA receptors. Changes to AMPA receptor subtype can be assessed by measuring AMPA receptor current using a -70/40 ratio or channel dynamics through IV plots. We identified no significant difference in the -70/40 ratio (Figure 3B) nor in rectification at positive potentials between adult and young animals (Figure 3C; +20 and +40 mv). This suggests AMPA receptor subunit composition remains similar across age groups. In addition, chronic THC injections in the young mice did not affect AMPA/NMDA or -70/40 mV ratios compared to control young mice (Figures 3A, B). We next examined input/output curves to assess AMPA receptor evoked current in adults and adolescents. This is explained in more detail in the methods section. There was no significant difference in AMPA currents between adult and young mice (Figure 3D). However, NMDA evoked current size is greater in young mice than adults (Figure 3E). Collectively, this suggests that the increased AMPA/NMDA receptor ratio in adults is less likely due to an increase in AMPA receptors, and more likely a result of decreased NMDA receptors.

**FIGURE 4:**
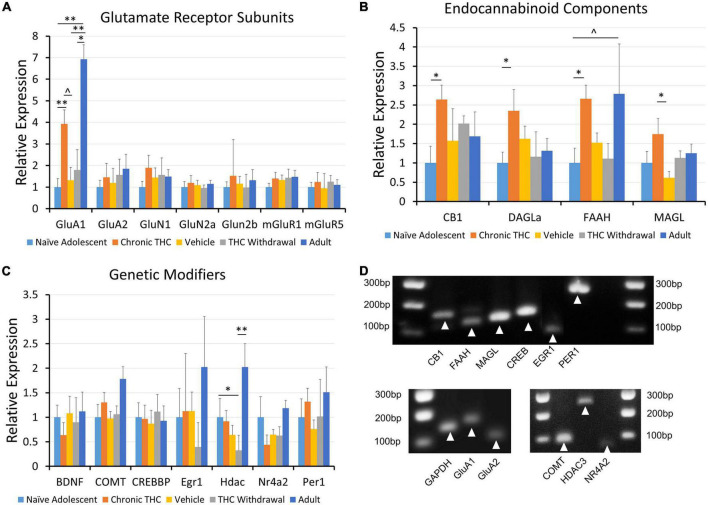
Expression of mRNA for CB1, GluA1, DAGLα and FAAH is modified following chronic THC injection or development. **(A)** Glutamate receptor subunits are largely unchanged across all groups, with the GluA1 subunit being a notable exception. GluA1 gene expression is increased following chronic THC exposure compared to naive young mice (*p* < 0.05). GluA1 gene expression is also increased in adults compared to naive young mice (*p* < 0.001), vehicle controls (*p* < 0.05), and THC withdrawal groups (*p* < 0.05). **(B)** Endocannabinoid components exhibit significant changes or trends following chronic THC injection. Gene expression for the CB1 receptor is significantly increased following chronic THC exposure compared to naive young mice (*p* < 0.05). DAGLα shows significant (*p* < 0.05) increased gene expression following chronic THC exposure, while FAAH demonstrates a trend in Naive adults compared to naïve young mouse levels (0.1 > *p* < 0.05). MAGL is increased in chronic THC exposed mice compared to vehicle control mice (*p* < 0.05). **(C)** Various genetic transcription or epigenetic modifiers were also examined, where HDAC3 gene expression decreased (*p* < 0.05) in young THC withdrawal groups when compared to naive young mice. HDAC3 gene expression is also increased in adults compared to THC withdrawal group (*p* < 0.05). **(D)** Proper amplification of each cellular target was examined using 4% agarose gel electrophoresis to verify amplicon size. See Table 1 for base pair (BP) lengths. Targets not shown in the gel were published previously (Merrill et al., 2015). The *n* value ranged from 7 to 10 for each group with at least 3 females and 3 males per group, and each *n* represents one mouse. The range was the result of some failed PCR experiments in some groups, indicated by qPCR results > 20 cycles from the control target, illustrating non-specificity, likely resulting from experimental error. *p < 0.05, ^**^p < 0.001.

The physiological and behavioral changes caused by drug exposure are often driven by changes in gene expression. Upon noting that chronic THC exposure creates long-lasting changes to VTA physiology, we employed quantitative RT-PCR to investigate changes in mRNA levels. Because we saw physiological differences between adult and young mice, age became a pertinent variable to assess as well. Note that these experiments use VTA punches that include all cell types within the VTA. Single cell PCR cannot accurately be used to examine quantitative changes in mRNA levels due to high level of false negatives that generate a 0 value, but are normally used to verify presence or absence of a target. Thus we used VTA punches versus single cells in order to more accurately quantify general changes in mRNA levels. Although our focus is broadened to include other cell types within the VTA, the transcriptional changes can still explain the physiological changes recorded in this study. For example, DA neurons produce endocannabinoids that could also regulate GABA cell synaptic activity. Thus we investigated differential mRNA expression for glutamate receptor subunits (Figure 4A), endocannabinoid components (Figure 4B) and epigenetic modifiers (Figure 4C). One element of drug of abuse is how changes in epigenetic or developmental regulating elements can be involved in dependence. This category will examine epigenetic changes and overall this data could provide targets for future investigation of chronic THC impact on VTA function. We examined targets from naive young mice (control), chronic THC-injected mice, vehicle-injected mice as a comparison with THC-injected, withdrawal of chronic THC and naive adult mice (Figure 4). Compared to naive young mice, GluA1 receptor subunits increased in both chronic THC exposed mice and naive adult mice. In addition, chronic THC-injected animals exhibited significant increases in CB1 receptor transcripts and fatty acid amide hydrolase (FAAH) mRNA transcripts. Importantly, after a 7 day withdrawal period, chronic THC treated mice were no longer significantly different from naive mice, suggesting THC-induced changes of mRNA are reversible similar to physiology. Lastly, chronic THC withdrawal treated mice showed a decrease in HDAC3 mRNA transcripts when compared to naive young mice (Figure 4C). Gel electrophoresis confirmed appropriate amplicon sizes (Figure 4D). Note that control naïve adults were examined in order to compare to control naïve adolescents.

**FIGURE 5:**
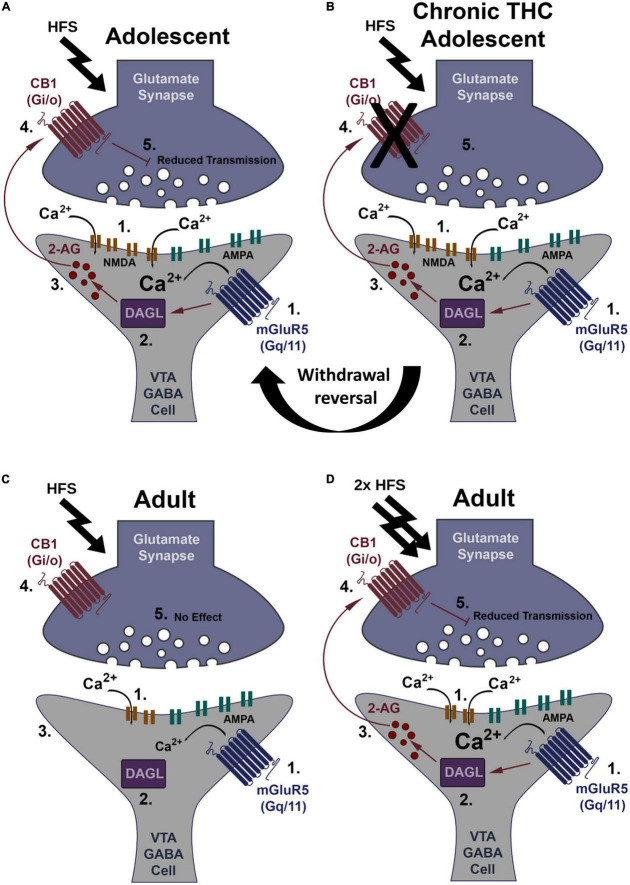
Working model of CB1-dependent LTD of VTA GABA neurons and increased induction thresholds in adults. **(A)** HFS (2 × 100 Hz) within young mice causes glutamate release sufficient to activate both mGluR5 and NMDA receptors, causing Ca^2+^ influx (1). Ca^2+^ activates DAGLα (2), creating the retrograde signaling endocannabinoid, 2-AG (3). 2-AG leaves the postsynaptic neuron and binds presynaptic CB1 receptors (4). CB1 activation depresses glutamate transmission resulting in LTD (5). **(B)** HFS within young mice chronically exposed to THC does not induce LTD. This loss of LTD is potentially at the CB1 receptor through internalization following constitutive activation by THC or impediments to its signal cascade. Following withdrawal this synapse reverses back to plasticity induction capacity similar to THC naïve (see “Withdrawal reversal”). **(C)** Within adults, HFS is not strong enough to induced LTD, likely due to a potential decrease in NMDA receptor levels reducing Ca^2+^ influx (1). Lower Ca^2+^ levels are insufficient to activate DAGLα (2). No 2-AG is produced (3), CB1 is not activated (4), and there is no LTD (5). **(D)** Double HFS within adults causes glutamate release sufficient to activate both mGluR5 and NMDA receptors more intensely, causing increased Ca^2+^ influx (1). Ca^2+^ activates DAGLα (2), creating the retrograde signaling endocannabinoid, 2-AG (3) that binds the CB1 receptor (4) to induce LTD (5).

In summary, chronic THC elimination of CB1 receptor-dependent LTD and depression is reversible after 7 days THC withdrawal. Additionally, AMPA/NMDA receptor ratio increases in adult rodents likely due to reduced NMDA current, suggesting a reduction in NMDA receptor levels could result in an increased induction threshold for adult LTD. The main findings are summarized in a working model (Figure 5).

## Discussion

CB1-dependent LTD of excitatory inputs to VTA GABA neurons is a form of synaptic plasticity conserved across species, suggesting its potential importance in the reward circuit. We show the elimination of LTD after chronic THC in young mice is fully reversible after withdrawal, suggesting THC-induced effects are not permanent. Finally, a new line of compelling data illustrate that adults require an increased induction stimulus compared to young mice to produce CB1-dependent plasticity, illustrating quantitative changes in CB1 forms of plasticity into adulthood.

### Mechanisms of THC-induced changes in CB1-dependent plasticity

THC is known to alter NMDA forms of plasticity, such as hippocampal LTP (Fan et al., 2010; Chen et al., 2013), but can also induce plasticity directly via CB1 receptor activation (Hoffman et al., 2007; Lupica et al., 2017), with the latter being our current focus. We recently reported a novel form plasticity in the VTA, where glutamatergic inputs to GABA neurons undergo a presynaptic form of LTD following HFS-conditioning. Here we confirm this LTD of VTA GABA neurons is conserved in rats. Conservation of this plasticity indicates it is shared among related species and not specific to mouse. Future studies would need to determine whether this plasticity is relevant to humans.

Our prior study also determined that chronic, but not a single, IP THC injections results in a loss of LTD. One goal now was to determine the mechanism of this elimination. CB1 agonist WIN55,212-2 induced depression of synaptic transmission in naïve mice (Friend et al., 2017). We determined that WIN55,212-2-induced depression was eliminated following chronic THC injection. This suggests synaptic CB1 receptors are either desensitized or removed following chronic THC injection, or that downstream signaling in the G_*i*_ pathway has been effected. Desensitization of CB1 by THC seems less likely however as these studies were performed 24 h after the last THC injection, and THC is not detectable in most brain regions 24 h post injection (Hoffman et al., 2007). That being said, changes noted in RNA levels for MAGL and DAGLα after chronic THC exposure suggest altered 2-AG metabolism as a possible explanation.

Many forms of drug-induced synaptic changes are reversible (Koob and Volkow, 2010). This reversal correlates with withdrawal symptoms, which manifest as feelings of unease, irritability or anxiety that can only be remedied by using the drug (Hyman and Malenka, 2001). Therefore, we examined whether LTD could return following one week of abstinence after chronic THC exposure. THC-induced synaptic changes in many brain areas are known to persist long-term (Hoffman et al., 2021), but the exact duration is unknown. Both HFS-induced LTD and WIN55,212-2-induced depression of excitatory inputs to VTA GABA neurons are restored one-week following THC withdrawal. This illustrates the ability and timeframe for synaptic plasticity to reverse following chronic THC exposure. This is an important consideration for dependence treatment and research purposes.

### Adult resistance to HFS-induced LTD

Significant alterations to plasticity occur during maturation into adulthood throughout the brain (Naveh-Benjamin, 2000; Cabeza et al., 2002). In addition, the negative impact of THC is more often seen in humans during adolescents than in adulthood, during which development of the endocannabinoid system is also changing (Ehrenreich et al., 1999; Pope et al., 2003; Gruber et al., 2014; Albertella et al., 2018; Hosseini and Oremus, 2019). This suggests that age-related differences in the eCB system could underlie differences in negative THC cognitive outcomes or in endocannabinoid-dependent plasticity. Adult mice over 70-days old exhibit quantitatively different LTD compared to young mice, but qualitatively similar because adults maintained CB1 expression, DHPG-induced depression, and LTD was still presynaptic. We also note that chronic THC exposure continues to eliminate LTD in adults as it does in young mice. We believe this is the first identification of changes in adult plasticity compared to young mice in the VTA, and is an important phenomenon in our understanding of the reward system and reward learning via synaptic plasticity. Therefore age should be considered as a variable in future VTA synaptic plasticity studies.

Lastly, AMPA/NMDA ratios are increased in adults. As this form of LTD requires NMDA receptor activation (Friend et al., 2021), a decrease in NMDA receptor expression is a potential rational for the quantitative increase in LTD induction threshold. Decreases in NMDA receptors in adults (Gonzales et al., 1991; Shankar et al., 1998; Dickstein et al., 2007; Bodhinathan et al., 2010), leading to increased plasticity induction thresholds (Barnes et al., 2000) have also been noted in the rat hippocampus. While this is the most likely explanation, we cannot rule out that decreased CB1 expression in adults could contribute to LTD induction differences, although CB1 agonists and THC induce similar levels of depression in both. It is possible that there is a floor effect in CB1 agonist-induced depression where fewer CB1 receptors in adults can still create maximum depression by agonists, but be less sensitive to endogenous eCB signals due to decreased receptor numbers *ex vivo*. Alternatively, differences in constitutive CB1 activation could be different as well between the two. Lastly, alterations to the endocannabinoid system as examined by qRT-PCR could also explain differences in plasticity induction in young versus adult mice, and is discuss below.

On a macro level, increased induction thresholds for VTA GABA neuron plasticity could provide a new hypothesis for why adults exhibit greater resistance to learning, anxiety, and addiction. Adults experience less anxiety and emotional intensity than children and adolescents (Allen and Matthews, 1997; Silvers et al., 2012; Bailen et al., 2019), which likely contributes to adolescent substance abuse (Spear, 2000). Additionally, THC and other drugs of abuse show reduced efficacy in adults (Chambers et al., 2003; Crews et al., 2007). Therefore, decreased readiness of GABA cells to undergo LTD and disinhibit DA neurons in adults would increase the stimulus necessary to promote these outcomes. For example, GABA neurons inhibit DA neuron activity (Bocklisch et al., 2013), and DA release is tied to addiction (Bouarab et al., 2019), reward driven learning/memory consolidation (Lisman and Grace, 2005; Berridge, 2007; Ghanbarian and Motamedi, 2013), and fear conditioning (De Oliveira et al., 2011, 2014; Bouarab et al., 2019). Indeed, because of their regulation of DA neurons, any cognitive effect tied to VTA DA release could be influenced by reduced GABA LTD in adulthood.

### THC- and age-induced changes to synaptic and endocannabinoid elements

Many drugs of abuse increase AMPA/NMDA ratios in DA neurons (Aitta-Aho et al., 2012; Ostroumov and Dani, 2018) as well as induce epigenetic or transcriptional changes believed to contribute to dependence (Robison and Nestler, 2011; Hurd and O’brien, 2018). Therefore, examining transcriptional changes caused by *in vivo* THC exposure in the VTA at large is necessary. As molecular changes in the endocannabinoid system could mediate differences in adult versus adolescent plasticity, qRT-PCR could help address this question. As these data are whole VTA homogenate, they correspond to all cells in the VTA, which provides a global perspective of chronic THC impact. For example, as endocannabinoid production in DA cells (Gardner, 2005) could also regulate CB1 receptor activation of GABA cell inputs, it provides a more detailed picture of changes in the eCB circuit as a whole and is therefore more relevant to this study. Our findings also provide targets for future investigation.

Regarding endocannabinoid components, qRT-PCR analysis of VTA slices from mice chronically exposed to THC displayed an increase in mRNA levels for FAAH. Because FAAH hydrolyses anandamide, this increase may lead to lower levels of anandamide after THC exposure. In the NAc of young mice, chronic THC similarly decreases anandamide (AEA) (Ellgren et al., 2008). We also noted an increase in VTA CB1 receptors mRNA transcripts (Figure 5B). While previous studies note decreased CB1 labeling and expression in the VTA following chronic THC exposure (Rubino et al., 2015; D’souza et al., 2016), others show differential CB1 mRNA expression based on brain area (Zhuang et al., 1998). Our results could suggest that increased CB1 mRNA is an adaptive response to counter THC-induced desensitization or endocytosis of CB1. Another interesting factor to note in young mice specifically is increased MAGL in THC exposed compared to THC withdrawal. This could lead to a decrease in 2-AG levels, making LTD more difficult to attain in chronic THC exposed adolescents, which supports our electrophysiology data. Lastly, differences in LTD between adults and adolescents due to increased 2-AG degradation or production by MAGL or DAGLα, respectively, are not supported by PCR data as there are no differences in young versus adult mice.

Our qRT-PCR results of significantly increased mRNA transcripts for GluA1 are consistent with reports regarding other drugs of abuse. For example, cocaine can increase VTA GluA1 mRNA expression in mice (Carlezon and Nestler, 2002) and cocaine, morphine or stress treatment can elevate protein expression of GluA1 in the mouse VTA (Lüscher and Malenka, 2011; Ostroumov and Dani, 2018). Our data add to this narrative, illustrating a similar pattern to other abused drugs, with THC exposure leading to an increase in GluA1 mRNA levels. Lastly, naïve adult mice showed a significant increase in GluA1 expression compared to young mice. Regulation of GluA1 expression is linked to many aspects of plasticity (Ge and Wang, 2021), suggesting that increased basal levels of GluA1 expression in naïve adults could be linked to changes in plasticity.

Notable changes in GluA1, CB1 and FAAH reversed following one week of drug withdrawal, suggesting THC-induced molecular changes are reversible. However, THC withdrawal mice have decreased histone deacetylase 3 (HDAC3) expression, suggesting an increase in gene transcription capacity during the withdrawal period. Given that little previous evidence has linked histone deacetylases to THC, the downregulation of HDAC3 transcripts seems to show a novel aspect of THC withdrawal, and is a great target of future examination. Histone deacetylases have been implicated in mitigating reward in other drugs of abuse. For example, inhibition of HDAC3 diminishes cocaine-seeking behavior (Malvaez et al., 2013) and histone deacetylase inhibitors can reverse morphine induced VTA synaptic plasticity (Authement et al., 2016). We also emphasize that we cannot make significant data interpretations for chronic THC if it was also not significantly different from chronic vehicle injections, even though trends appeared. Lastly, it is important to note that qRT-PCR mRNA findings do not necessarily correlate to direct protein expression (Vogel et al., 2010), however they do provide evidence for relevant future targets to examine as we have demonstrated previously (Miller et al., 2018). To our knowledge, we are the first to examine VTA mRNA expression of endocannabinoid synthesizing and metabolizing enzymes in mice following chronic THC exposure and development into adulthood.

## Data availability statement

The original contributions presented in this study are included in the article/Supplementary material, further inquiries can be directed to the corresponding author.

## Ethics statement

The animal study was reviewed and approved by Brigham Young University Institutional Animal Care and Use Committee and Animal Welfare Assurance Number: A3783-01.

## Author contributions

IO and JE designed the experiments. IO and MV acquired and analyzed the electrophysiological data. IO, MV, and JE wrote the manuscript. CS acquired and analyzed the PCR data and helped to write and edit the manuscript. All authors contributed to the article and approved the submitted version.
