# Supplementary material for: Chronic Δ9-tetrahydrocannabinol impact on plasticity, and differential activation requirement for CB1-dependent long-term depression in ventral tegmental area GABA neurons in adult versus young mice
Source: Front Neurosci. 2023 Jan 9;16:1067493. doi: 10.3389/fnins.2022.1067493 (PMC9869137; doi:10.3389/fnins.2022.1067493)
Supplement: Supplementary file 1 [file Data_Sheet_1.docx]

**Supplemental Data**


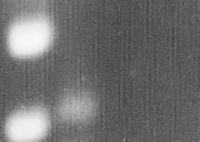


GAD67

ACSF+GAD67

TH

150bp

100bp

A

B

*Supplemental Figure 1.* ***High-frequency stimulus*** *(****HFS)-induced LTD in young rodents.*** ***A,*** *In* *VTA GABA neurons of naïve young rats (P23-45) HFS stimulus induced significant LTD (24.6 ± 8.6% reduction) at 15-20 min and (30 ± 9.3%) 35-40 min post stimulus compared to baseline (n=4, p = 0.000009 and p = 0.000008). Plot is of whole-cell normalized excitatory post-synaptic currents (EPSC) amplitude means with bars indicating standard error of the mean (SEM). Arrow indicates time of HFS. Example traces representing 12-14 averaged traces before (black) and 15-20 min after (grey) conditioning or drug application. Note that all stimulation artifacts were removed from example traces. Scale bars represent 50 pA, 5 ms.* ***B,*** *Quantitative PCR fluorescent curve of a representative recorded rat GABAergic cell expressing GAD67, but lacking TH. 18S is the housekeeping control measure of baseline expression. Relative fluorescence units (RFU) were generated by Evagreen or probe and graphed relative to the number of PCR cycles performed. Inset top: Gel electrophoresis from a PCR reaction demonstrating single cell expression of GAD67 within a rat GABA neuron. Note the lack of TH amplicon and also the absence of GAD67 in the background control for GAD67 (ACSF+GAD67). Amplicon sizes for PCR products are TH: 108bp; GAD67: 101bp. Inset bottom: Representative hyperpolarization trace induced by -200 pA. GABA neurons do not exhibit significant I_h_ currents as noted here.*

A

B

*Supplemental Figure 2.* ***NMDG cutting solution does not influence plasticity induction in adults.*** ***A.*** *Young mice cut and perfused with NMDG cutting solution continue to exhibit the same LTD induction following HFS as seen in sucrose cutting solution (30.3 ± 6.8% LTD; n=6, p < 0.001 compared to baseline, p > 0.5 compared to Figure 1A).* ***B.*** *Adult mice cut in sucrose solution do not exhibit LTD following HFS, as also seen in adult NMDG cut slices (n=5, p > 0.5 compared to baseline, p > 0.5 compared to 3.*

| Within Experiment and Between Group Statistical Analysis Summary | | | |
| --- | --- | --- | --- |
| Group(s) | **Test** | **P Value** | **Location** |
| Naïve Young Mice BL to Post-HFS | ANOVA | p < 0.001 | Figure 1A |
| Young Mice Chronic THC BL to Post-WIN55,212-2 | ANOVA | p > 0.1 | Figure 1B |
| Young Mice Chronic Vehicle BL to Post-WIN55,212-2 | ANOVA | p < 0.001 | Figure 1C |
| Young Mice 7 Days THC Withdrawal BL to Post-WIN55,212-2 | ANOVA | p < 0.001 | Figure 1D |
| Young Mice Chronic THC Post-WIN55,212-2 to Young Mice 7 Days THC Withdrawal Post-WIN55,212-2 | Unpaired two-way T-test | p < 0.001 | Figure 1D |
| Young Mice 7 Days THC Withdrawal BL to Post-HFS | ANOVA | p < 0.001 | Figure 1E |
| 7 Days Withdrawal from Chronic Vehicle Young Mice BL to Post-HFS | ANOVA | p < 0.001 | Figure 1F |
| Naïve Adult Mice BL to Post-HFS | ANOVA | p > 0.1 | Figure 2A |
| Naïve Adult Mice BL to Post WIN55,212-2 | ANOVA | p < 0.001 | Figure 2B |
| Naïve Adult Mice Post-HFS to Naïve Adult Mice Post-WIN55,212-2 | Unpaired two-way T-test | p < 0.001 | Figure 2B |
| Naïve Adult Mice BL to Naïve Adult Mice Post-DHPG | ANOVA | p < 0.001 | Figure 2C |
| Naïve Adult Mice Post-HFS to Naïve Adult Mice Post-DHPG | Unpaired two-way T-test | p <0.001 | Figure 2C |
| Naïve Adult Mice Post-DHPG to Naïve Young Mice Post-DHPG (Previously reported) | Unpaired two-way T-test | p > 0.1 | Figure 2C & Friend 2017 |
| Naïve Adult Mice Post-WIN55,212-2 to Naïve Young Mice Post-WIN55,212-2 (Previously reported) | Unpaired two-way T-test | p > 0.1 | Figure 2B & Friend 2017 |
| Naïve Adult Mice BL to Naïve Adult Mice Post-2xHFS | ANOVA | p < 0.001 | Figure 2D |
| Naïve Adult Mice Post-HFS to Naïve Adult Mice Post-2xHFS | Unpaired two-way T-test | p < 0.001 | Figure 2D |
| Naïve Adult Mice BL to Naïve Adult Mice Post-2xHFS with AM251 | ANOVA | p > 0.1 | Figure 2E |
| Naïve Adult Mice HFS to Naïve Adult Mice Post-2xHFS with AM251 | Unpaired two-way T-test | p > 0.1 | Figure 2E/A |
| Adult Chronic THC BL to Post-2xHFS | ANOVA | p > 0.1 | Figure 2F |
| Adult Chronic THC Post-2x-HFS to Naïve Adult Post 2xHFS | Unpaired two-way T-test | p < 0.001 | Figure 2F |
| Naïve Adult Coefficient of Variance BL to Post-2xHFS | Wilcoxon Rank Sum | p < 0.05 | Figure 2H |
| Naïve Adult Coefficient of Variance BL to Post-WIN55,212-2 | Wilcoxon Rank Sum | p < 0.05 | Figure 2H |
| Naïve Adult Coefficient of Variance BL to Post-DHPG | Wilcoxon Rank Sum | p < 0.05 | Figure 2H |
| Naïve Adult Paired Pulse Ratio BL to Post-2xHFS | Wilcoxon Rank Sum | p < 0.05 | Figure 2I |
| Naïve Adult Paired Pulse Ratio BL to Post-WIN55,212-2 | Wilcoxon Rank Sum | p < 0.05 | Figure 2I |
| Naïve Adult Paired Pulse Ratio BL to Post-DHPG | Wilcoxon Rank Sum | p < 0.1 | Figure 2I |
| AMPA/NMDA Ratio Naïve Adult to Naïve Young | Unpaired two-way T-test | p < 0.05 | Figure 3A |
| AMPA/NMDA Ratio Naïve Young to Chronic THC Young | Unpaired two-way T-test | p > 0.1 | Figure 3A |
| -70/40 Ratio Naïve Adult to Naïve Young | Unpaired two-way T-test | p > 0.1 | Figure 3B |
| -70/40 Ratio Naïve Young to Chronic THC Young | Unpaired two-way T-test | p > 0.1 | Figure 3B |
| IV Plots Naïve Young to Naïve Adult | ANCOVA | p > 0.1 | Figure 3C |
| Input/Output Curves of Evoked AMPA Currents by Stimulation Intensity Naïve Young Mice to Naïve Adult Mice | ANCOVA | p > 0.1 | Figure 3D |
| Input/Output Curves of Evoked NMDA Currents by Stimulation Intensity Naïve Young Mice to Naïve Adult Mice | ANCOVA | p < 0.05 | Figure 3E |
| GluA1 Subunit mRNA Relative Expression Naïve Young Mice to Chronic THC Young Mice | ANOVA & Tukey Post-Hoc | p < 0.05 | Figure 4A |
| GluA1 Subunit mRNA Relative Expression Naïve Adult Mice to Naïve Young Mice | ANOVA & Tukey Post-Hoc | p < 0.001 | Figure 4A |
| GluA1 Subunit mRNA Relative Expression Naïve Adult Mice to Chronic Vehicle Young Mice | ANOVA & Tukey Post-Hoc | p < 0.05 | Figure 4A |
| GluA1 Subunit mRNA Relative Expression Naïve Adult Mice to THC Withdrawal Young Mice | ANOVA & Tukey Post-Hoc | p < 0.05 | Figure 4A |
| CB1 Receptor mRNA Relative Expression Naïve Young Mice to Chronic THC Young Mice | ANOVA & Tukey Post-Hoc | p < 0.05 | Figure 4B |
| CB1 Receptor mRNA Relative Expression Naïve Young Mice to THC Withdrawal Young Mice | ANOVA & Tukey Post-Hoc | p > 0.1 | Figure 4B |
| DAGLα mRNA Relative Expression Naïve Young Mice to Chronic THC Young Mice | ANOVA & Tukey Post-Hoc | p < 0.05 | Figure 4B |
| FAAH mRNA Relative Expression Naïve Adult Mice to Naïve Young Mice | ANOVA & Tukey Post-Hoc | P < 0.1 | Figure 4B |
| MAGL mRNA Relative Expression Chronic THC Young Mice to THC Withdrawal Young Mice | ANOVA & Tukey Post-Hoc | p < 0.05 | Figure 4B |
| HDAC3 mRNA Relative Expression Chronic THC Young Mice to THC Withdrawal Young Mice | ANOVA & Tukey Post-Hoc | p < 0.05 | Figure 4C |
| HDAC3 mRNA Relative Expression Naïve Adult Mice to THC Withdrawal Young Mice | ANOVA & Tukey Post-Hoc | p < 0.05 | Figure 4C |

*Supplemental Table 1.* ***Statistical analysis and significance of experimental data.***
